# Discovery of a PFKFB3 inhibitor for phase I trial testing that synergizes with the B-Raf inhibitor vemurafenib

**DOI:** 10.1186/2049-3002-2-S1-P14

**Published:** 2014-05-28

**Authors:** Sucheta Telang, Julie O’Neal, Gilles Tapolsky, Brian Clem, Alan Kerr, Yoannis Imbert-Ferndandez, Jason Chesney

**Affiliations:** 1 JG Brown Cancer Center, University of Louisville, Louisville, KY, USA; 2 Department of Biochemistry, University of Louisville, Louisville, KY, USA; 3 Advanced Cancer Therapeutics, Louisville, KY, USA

## Background

In human cancers, loss of PTEN, stabilization of hypoxia inducible factor-1α, and activation of Ras and AKT converge to increase the activity of a regulator of glycolysis, 6-phosphofructo-2-kinase/fructose-2,6-bisphosphatase (PFKFB3). This enzyme synthesizes fructose-2,6-bisphosphate (F2,6BP), which is an activator of 6-phosphofructo-1-kinase, a key step of glycolysis that is tightly controlled by multiple metabolic feedback mechanisms. We recently identified the first competitive small molecule inhibitor of PFKFB3, 3-(3-pyridinyl)-1-(4-pyridinyl)-2-propen-1-one (3PO), and have now sought to develop a more potent PFKFB3 inhibitor with improved PK properties for testing in clinical trials.

## Materials and methods

Methods included recombinant PFKFB3 assays, PK studies using mass spectrometry, pre-clinical toxicity and efficacy studies, Western blot analyses for HIF-1α and PFKFB3 in A375 melanoma cells, F2,6BP assessments and flow cytometry for apoptosis.

## Results

We report the discovery of a novel 3PO-derivative, PFK158, that is more potent than 3PO, has improved PK properties and causes ~80% growth inhibition in several mouse models of human-derived tumors. We also demonstrate that PFK158 is well tolerated in rats and dogs providing key support for a phase 1 trial in cancer patients that will initiate in 2014. Once the MTD is established, we intend to conduct multiple phase 1/2 trials of PFK158 in combination with targeted agents given the ability of PFK158 to suppress glycolysis. 50% of melanomas harbor a BRAF^V600E^ mutation that promotes glucose metabolism, survival and proliferation and BRAF^V600E^ inhibitors are effective in ~50% of melanoma patients. Unfortunately, resistance to these agents develops within six months and most patients die within two years of diagnosis. Genetic amplifications of BRAF^V600E^ are a common cause of resistance, and BRAF^V600E^ stabilizes HIF-1α, an established promoter of PFKFB3. We thus hypothesized that PFKFB3 may be essential for intrinsic resistance to BRAF^V600E^ inhibitors. In unpublished results, we demonstrate that 3 hours of exposure to 1 μM vemurafenib reduces HIF-1α, PFKFB3, and F2,6BP in BRAF^V600E^ positive A375 melanoma cells and that PFK158 markedly sensitizes these cells to the apoptotic effects of vemurafenib.

## Conclusions

In conclusion, PFK158 is the first PFKFB3 inhibitor to be examined in a phase I trial and may have significant clinical utility when combined with agents that target driver oncogenes. Importantly, our data provide rationale for the conduct of pre-clinical studies of PFK158 combined with vemurafenib in transgenic BRAF^V600E^ melanoma mouse models which are in turn expected to justify a phase 1/2 trial of the combination in BRAF^V600E^-positive melanoma patients.

